# Evaluation of Antimicrobial and Antibiofilm Activities of Copper Oxide Nanoparticles within Soft Denture Liners against Oral Pathogens

**DOI:** 10.1155/2021/9939275

**Published:** 2021-06-04

**Authors:** Elham Ansarifard, Zahra Zareshahrabadi, Najmeh Sarafraz, Kamiar Zomorodian

**Affiliations:** ^1^Department of Prosthodontics, School of Dentistry, Shiraz University of Medical Sciences, Shiraz, Iran; ^2^Nanobiology and Nanomedicine Research Center, Shiraz University of Medical Sciences, Shiraz, Iran; ^3^Department of Parasitology and Mycology, School of Medicine, Shiraz University of Medical Sciences, Shiraz, Iran; ^4^Department of Periodontology, School of Dentistry, Rafsanjan University of Medical Sciences, Rafsanjan, Iran; ^5^Basic Sciences in Infectious Diseases Research Center, Shiraz University of Medical Sciences, Shiraz, Iran

## Abstract

**Objectives:**

Soft denture liners provide a favorable environment for adhesion and colonization of microorganisms. This in vitro study aimed to examine the efficacy of different concentrations of copper oxide nanoparticles (CuO NPs) incorporation into soft denture liner on the biofilm formation of the microbial species.

**Methods:**

Field Emission Scanning Electron Microscopy (FESEM) images from NPs were recorded. Antifungal susceptibility testing of CuO NPs against five standard strains of *Candida albicans* (CBS 10261, 1905, 1912, 1949, 2730), *Streptococcus mutans* (ATCC35668), *Streptococcus sobrinus* (ATCC27607), and *Streptococcus salivarius (*ATCC9222) was performed by the broth microdilution method with the Clinical and Laboratory Standards Institute reference method. The biofilm inhibition percentages of CuO NPs on the soft denture liners were determined by XTT assay.

**Results:**

The characterization of CuO NPs by scanning electron microscope (SEM) analyses confirmed the synthesis of NPs with appropriate structure and size with a mean diameter of 18.3 ± 9.1 nm. The CuO NPs successfully inhibited the growth of the tested standard strains of *C. albicans a*nd *Streptococcus* spp. at concentrations ranging from 64 to 128 *µ*g mL^−1^. Indeed, incorporation of CuO NPs at a concentration of 500 *µ*g mL^−1^ into the soft denture liners exhibited a significant activity (75%) in inhibition of *C. albicans*. biofilm formation in a dose-dependent manner. The biofilm formation of *C. albicans* in the presence of CuO NPs was lower than *Streptococcus* spp. in comparison with the control group (*p* < 0.05).

**Conclusion:**

Incorporation of CuO NPs significantly decreased the colonization and plaque formation of the oral pathogens, especially *C. albicans* accumulation. These NPs may be useful as a promising agent for the antimicrobial management of soft denture liner materials.

## 1. Introduction

Soft denture liners are resilient materials that are used as a shock absorber under the denture, to reduce the functional loads to the bearing mucosa by distributing the loads homogeneously on the denture-bearing tissues [1]. This is mainly useful in patients with sharp or atrophic ridges having a low tolerance to the loads applied by the dentures. They are also commonly used for conditioning the irritated tissues mostly induced by ill-fitted dentures. Soft denture liners are prone to easy deterioration, which increases the surface roughness. It will result in an increase in susceptibility to colonization and growth of the microorganisms. Studies have shown that fungal and bacterial microorganisms initially adhere to the surface of the lining and accumulations on the appliances [2–4]. In the oral cavity, most colonizing and infecting microorganisms are found not as single-living cells, but rather as complex structured microbial communities, often encapsulated within a matrix of exopolymeric material, and attached to biotic or abiotic surfaces. These communities are referred to as biofilms [5, 6]. Microbial biofilms are 3D structures that have gained the ability to resist antimicrobials and immune cell challenges. It is noteworthy that environmental conditions under the denture as well as the structure of materials supported this microbial growth and played a key role in denture-related stomatitis [7, 8]. Denture stomatitis is a common disease in denture wearers (15–70%) which mainly contributes to *Candida* spp. and oral bacteria [6, 9]. On the other hand, for some elderly patients with some physical and mental disabilities, conventional chemical and mechanical denture cleansing procedures are challenging [10].

Moreover, some of the mentioned cleansing methods can also damage the soft denture liners and cause more microbial plaque accumulation [11, 12]. Several researchers have already incorporated a variety of medicament including antifungal drugs, metal oxides, and herbs into the soft denture liners, as a reliable method to overcome denture-induced stomatitis [13, 14]. Although many of these modified soft denture liners showed promising results against microorganisms, different shortcomings were also reported for the investigated additives. For example, there was no certain durability and robustness of the effective dose for the evaluated additives. Some of these soft denture liners were reported to be effective only for a short time (3–7 days) [4, 15]. Some other studies demonstrated that some of these antifungal additives compromise the mechanical properties of the soft denture liners [16, 17]. Although the beneficial effect of antimicrobial additives to the soft denture liners has been proved, no antimicrobial soft liner is commercially available, yet. Nanotechnology has numerous modern applications in the field of dentistry, including disease diagnosis, therapy, and prevention. Restorative dentistry, cancer diagnosis and treatment, implant dentistry, and molecular imaging are some examples of the application areas [18]. Nanomaterials are natural or manufactured materials containing particles in the size ranges of 1–100 nm [19]. Recently, copper oxide nanoparticles (CuO NPs) have been extensively used in different areas of science and technology due to their good thermal, electrical conductivity, and low cost [20]. Previous studies have demonstrated that the incorporation of NPs in orthodontic brackets and alginates significantly reduces the amount of microbial biofilm [21, 22]. Recently, in order to reduce both the cost and the toxicity of CuO NPs, biological sources such as plants have been used instead of chemical methods, which have shown an additional antibacterial activity, as well [23–25].

To the best of our knowledge, there is still no report about the antimicrobial and antibiofilm activity of the CuO NPs incorporated into the soft liner in different concentrations. Therefore, this study aimed to evaluate the effectiveness of incorporating CuO NPs into the soft liner and assess its antimicrobial and antibiofilm activity effects against *Streptococcus mutans*, *Streptococcus sobrinus, Streptococcus salivarius*, and *Candida albicans*.

## 2. Materials and Methods

### 2.1. Synthesis and Characterization of CuO NPs

Briefly, 25 mL ethanolic solution of copper acetate (Merck, Germany, 0.2 mol L^−1^) was mixed with 25 mL ethanolic solution of sodium hydroxide (Merck, Germany, 0.4 mol L^−1^) by a magnetic stirrer followed by the addition of 0.5 g polyethylene glycol (molecular weight, 20,000**)** (Merck, Germany). The mixture was then refluxed for 25 min. A brown precipitate was obtained that was washed several times by absolute ethanol and acetone. The sample (CuO NPs) was dried at room temperature. Besides, Field Emission Scanning Electron Microscopy (FESEM) images from NP_S_ were recorded using a TESCAN Mira 3-XMU (Czech Republic).

### 2.2. Preparation of Resin Disks Containing Different Concentrations of CuO NPs

In this study, we used a self-cured acrylic-based soft liner (GC Cooperation, Tokyo, Japan) which consists of powder and liquid. According to the manufacturer's instructions, experimental soft denture liner discs (10.0 × 3.0 mm) were fabricated containing 0, 0.5, 5, 50, and 500 *µ*g mL^−1^ concentrations of CuO NPs. After CuO NPs incorporation, the prob sonication (soniprep-150, England) was used for 3 minutes to disperse the mixture. Afterward, the powder and liquid were mixed placed in a disk-shaped silicone mold, pressed between two glass slabs until the soft denture liner cured, and then polished. A total of 80 discs were prepared and divided into five groups (*n* = 16) according to the concentration of CuO NPs incorporated. Then, within a group, four soft denture liner discs were assigned to each strain.

### 2.3. Disinfection of the Specimens

The specimens were disinfected by immersing in glutaraldehyde 2% for 2 min and rinsed with sterile water.

### 2.4. Antimicrobial Susceptibility Tests

The antimicrobial activity of CuO NPs against five standard strains of *Candida albicans* (CBS 10261, 1905, 1912, 1949, 2730), *Streptococcus mutans* (ATCC35668), *S. sobrinus* (ATCC27607), and *S. salivarius* (ATCC9222) was determined. Standard strains of bacteria and *C. albicans* were seeded on the brain heart infusion agar and Sabouraud dextrose agar (Merck, Germany), respectively. Then, the culture plates were incubated at 35 ± 2°C between 18 and 24 h. Minimum inhibitory concentration (MIC) of CuO NPs against standard strains of bacteria and *C. albicans* was determined by the broth microdilution method as recommended by the clinical and laboratory standards institute (CLSI), in the range of 0.5 *μ*g mL^−1^–512 *μ*g mL^−1^.

Briefly, RPMI-1640 medium (with L-glutamine and phenol red, without bicarbonate, Sigma, USA) was prepared and buffered at pH 7.0 with 0.165 mol 3-(N-morpholino) propane sulfonic acid (MOPS) (Sigma-Aldrich, Steinheim, Germany). Serial dilutions of NPs (0.5 to 512 *µ*g mL^−1^) were prepared in 96-well microtitre trays using RPMI-1640 buffered with MOPS. Stock inoculums were prepared by suspending three colonies of the examined bacteria and *C. albicans* in 5 mL sterile 0.85% NaCl and adjusting the turbidity of the inoculums to 0.5 McFarland standards at 530 nm wavelengths (this yields stock suspension of 1–5 × 10^6^ cells mL^−1^). Working suspension for *C. albicans* and bacteria was prepared by making 1/1000 and 1/100 dilutions, respectively, with broth media of the stock suspension. After the addition of 0.1 mL of the *C. albicans* and bacteria inoculums to the wells, the trays were incubated at 30°C for 24 h in a humid atmosphere. The uninoculated medium was included as a sterility control (blank). Besides growth controls, media with inoculums but without NPs were included. The growth in each well was compared with that of the growth control well. MICs were visually determined and defined as the lowest concentration of NPs that completely inhibited the growth of standard strains of bacteria and *C. albicans* [8].

### 2.5. Determining the Antibiofilm Activity

#### 2.5.1. Biofilm Preparation and Growth

For biofilm development, *C. albicans* (CBS 10261) strains were cultured in Sabouraud dextrose agar (Merck, Germany) medium while S. *mutans* (ATCC35668), *S. sobrinus* (ATCC27607), and *S. salivarius* (ATCC9222) were cultured in the brain heart infusion (BHI) (Merck, Germany) agar. After 24 h, one loop of *C. albicans* and bacteria strains colonies were transferred to 20 mL Sabouraud dextrose and brain heart infusion broth (Merck, Germany), respectively, and incubated at 30°C overnight in an orbital shaker (shaken at 100 rpm). *Candida albicans* and bacteria cells were then harvested and washed twice in sterile phosphate-buffered saline (PBS, 0.8% w/v), sodium chloride (0.02% w/v, Merck, Germany), KH_2_PO_4_ (0.31% w/v, Merck, Germany), Na_2_HPO_4_.12H_2_O (0.02% w/v, Merck, Germany), and KCl (Panreac, Madrid, Spain). Afterwards, a suspension of respective microorganisms was prepared in RPMI-1640 medium buffered with MOPS [3-(N-morpholino) propane sulfonic acid]. *C. albicans* stocks were used to prepare microbial suspension with 0.15 optical densities at 530 nm. This turbidity is equal to 1 × 10^5^ yeasts mL^−1^. All bacteria suspension with an optical density of 0.1 at 530 nm was also prepared. This turbidity is equal to 1.5 × 10^8^ CFU mL^−1^. Soft denture liner disks containing different concentrations of CuO NPs were then placed as pairs in sterile 12-well cell culture plates. One mL of microbial suspension was added to each well; then, the plates were placed at 30°C for 48 h to form a biofilm [26].

#### 2.5.2. Assessing Biofilm Formation

Biofilm formation was assayed by using a 2,3-bis(2-methoxy-4-nitro-5-sulfo-phenyl)-2H-tetrazolium-5-carbox-anilide (XTT, Sigma, USA) reduction assays. XTT was prepared as a saturated solution at a concentration of 0.5 mg mL^−1^ in Ringer's lactate. This solution was filter-sterilized through a 0.22 *µ*m pore size filter, divided into aliquots, and then stored at –70°C. Prior to each assay, an aliquot of the XTT stock solution was thawed, and treated with menadione sodium bisulfite (Sigma, USA, 10 mmol L^−1^ prepared in distilled water) to obtain the final concentration of 1 *µ*mol L^−1^ of menadione. After 48 h, the plates were brought out of the incubator, and the contents of each well were emptied. Plates were washed with PBS three times for the unattached microorganisms to be extracted from the plates. In the next phase, acrylic discs were gently transferred to new plates. A 500 *µ*L aliquot of XTT-menadione was then added to each pre-washed biofilm and the wells that contained a disk treated with CuO NPs and incubated for 4 h at 35°C in dark to measure the background XTT levels. Finally, the content of the wells was transferred to another plate and their spectral absorbance at a wavelength of 570 nm was evaluated by a multi-well scanning spectrophotometer (Polar star omega, Germany). All trials were performed in duplicate to reduce the error rate as much as possible. The well containing soft liner disks and culture medium without microorganism was considered as the negative control and soft liner disks in the wells containing media and organism were used as the positive control [26].

### 2.6. Statistical Analysis

Statistical analysis was performed using SPSS software (version 11.0) and Mann–Whitney *U* test was used to compare the microbial biofilm formation between the control and each tested concentrations groups. The quantitative data were presented in terms of mean and standard deviations and *p* value <0.05 was considered as the significance level threshold.

## 3. Results

**Figure 1:**
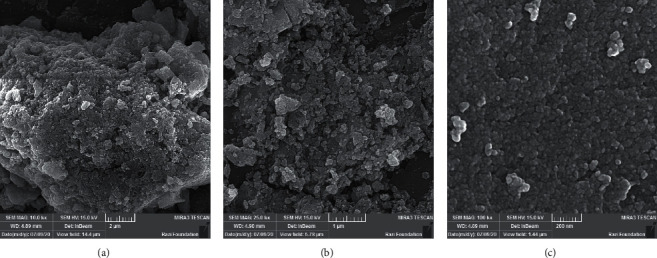
FESEM images of CuO NPs.

Characterization of CuO NPs by SEM analyses confirmed the synthesis of NPs with appropriate structure and size.  Figure 1 shows FESEM images recorded from CuO NPs at different magnifications. The lumps of the particles are observed in the low-magnified image, and at higher magnifications, the adhered NPs of CuO are observed. A mean diameter of 18.3 ± 9.1 nm (*n* = 50) was obtained for NPs.

Antifungal and antimicrobial test results are presented in Table 1. CuO NPs successfully inhibited the growth of the tested standard strains of *C. albicans* and bacteria at concentrations ranging from 64 to 128 *µ*g mL^−1^ (geometric mean = 84.44 *µ*g mL^−1^).

Moreover, biofilm formation of *C. albicans* (CBS 10261) and *Streptococcus* spp. in the presence of CuO NPs with 0.5, 5, 50, and 500 *µ*g mL^−1^ were measured quantitatively by the XTT reduction assay and the results are presented in Table 2.

The results indicated that CuO NPs exhibited a significant activity in inhibition of microbial biofilm formation in a dose-dependent manner, as reflected by lower absorbance reading when compared with the untreated control. Indeed, the *Candida* biofilm formation was inhibited by up to 75% at the concentration of 500 *µ*g mL^−1^ of CuO NPs. The biofilm formation of *C. albicans* in the presence of CuO NPs was significantly lower than *Streptococcus* spp. biofilm formation with a statistically significant difference (*p* < 0.05).

## 4. Discussion

Copper oxide is one of the copper compound families that exhibit useful physical properties, such as high-temperature superconductivity, electron correlation effects, and spin dynamics. It is relatively cheap, easily mixed with polar liquids and polymers, and comparatively stable in terms of chemical and physical activities. It is notable that the considerable antimicrobial effect of CuO NPs was achieved in previous studies [27, 28].

Intrinsic porosity in the soft denture liners makes them susceptible to be colonized by microorganisms. Microorganisms form a biofilm surface over the prosthesis in contact with oral tissue and may manifest as cracks and irregularities that occur in some stages of soft denture liner preparation. These defects can act as oral reservoirs for adhesion and colonization of microorganisms, which include *C. albicans* and *Streptococcus* species particularly*, S. mutans, S. sobrinus,* and *S. salivarius* [29, 30].

*Candida albicans* has the most important and predominant oral fungal pathogen. It can adhere and proliferate upon both soft and hard tissue surfaces within the oral cavity, and form biofilm. *Streptococcus mutans* is a major pathogen of dental caries that can cause significant health problems for denture wearers [26].

Due to some disadvantages of direct use of NPs including the objectionable taste of some additives and the necessity of frequent utilization that needs patient cooperation [31], the purpose of the present study was to assess the antimicrobial and antibiofilm effects of CuO NPs (in the mean diameter of 18.3 ± 9.1 nm) incorporated into soft denture liners.

In this study, CuO NPs inhibited the growth of *C. albicans* and oral *Streptococcus* spp. at a geometric means of 84.44 *µ*g mL^−1^. It is noteworthy that the microbial inhibition efficacy of NPs depends on their concentration as well as their size and shape. Khan et al. found that CuO NPs at 50 *μ*g mL^−1^ concentration significantly prevented the growth of some oral bacteria [32] and the MIC values of this study were comparable to the present study. Sathiyavimal et al. showed that non-toxic chitosan coated CuO NPs have a good inhibitory effect against specially Gram-negative bacteria rather than Gram-positive ones, at the concentration of 20–80 *µ*g mL^−1^ [24].

In this work, the antibiofilm activity of CuO NPs exhibited various effects against different microorganisms. In the current study, incorporating the CuO NPs in soft denture liner samples inhibited the biofilm formation of *C. albicans* and oral *Streptococcus* species in a dose-dependent manner. The bioactivity and biofilm formation of *C. albicans* and *Streptococcus* spp. successively decreased with increasing CuO NPs concentration by using XTT reduction assays. The MIC and biofilm formation results can probably explain the anti-adhesion and subsequently the antibiofilm effects of CuO NPs.

In all four concentrations of 0.5, 5, 50, and 500 *µ*g mL^−1^ of CuO NPs that were incorporated in soft denture liners, antibiofilm effects were shown in comparison to the control group, but it was not significant at lower concentrations of 0.5 and 5 *µ*g mL^−1^.

It seems that incorporating CuO NPs in low concentrations probably does not release and interfere in adhesion and biofilm formation. Based on our findings, there was 75% *C. albicans* biofilm inhibition at 500 *μ*g mL^−1^ of CuO NPs concentration. These findings were in the same line as those of Sivaraj et al. [33].

Among the bacterial species, *S. sobrinus* had the lowest rate (37%) of biofilm formation at 500 *μ*g mL^−1^ of CuO NPs, while *S. mutans* and *S. salivarius* were less susceptible species to CuO NPs (70% and 40% biofilm formation at 500 *μ*g mL^−1^, respectively).

The structure of the cell wall plays an important role in the tolerance or susceptibility of the microorganism in the presence of metal NPs. Based on previous findings, *Streptococcus* strains, as Gram-positive bacteria have a large cell wall, with multiple layers of peptidoglycan, and other protective surface structures [34, 35].

Based on the current study, soft denture liner containing 0.5, 5, and 50 *μ*g mL^−1^ CuO NPs had no significant effect on *Streptococcus spp.* adhesion and, subsequently, biofilm formation (*p* value > 0.05). These findings were in contrast with Eshed et al. study, indicating that CuO NPs at low concentration prevented the biofilm formation of *S. mutans* [36]. The possible explanation for these differences may be related to biofilm formation assay. In the present study, the XTT method was used as a quantitative measurement of biofilm formation.

The results of this microbial assay confirmed that the susceptibility of *C. albicans* to CuO NPs of the soft denture liner samples was more than oral *Streptococcus* strains. These findings were consistent with Méndez-Serrano's study [37].

In another study, the evaluation of *candidal* biofilm formation on the teeth coated with CuO NPs revealed the reduction of biofilm formation by 70% which is in line with the result of the present study [36]. In a study by Pugazhendhi et al., it was shown that Fe doped CuO has a very good antimicrobial and antibiofilm activity against *S. aureus, S. epidermidis*, and *C. albicans.* Due to its cationic nature, it would have promoted an easy binding and penetration, which leads to a fungicidal activity [38].

As revealed in another study, coated brackets with metal NPs showed dramatic antimicrobial and antibiofilm effects in comparison with the control groups [21]. The antimicrobial mechanisms of CuO NPs may be related to microbial cell wall attachment, and cause structural changes, which then provokes intracellular oxidative stress. Consequently, they reduce the vital activity of the microbial cell such as permeability, which then results in less biofilm formation activity and eventually leads to the death of the pathogens.

In a study, Tabrez Khan et al. found that 50 *μ*g mL^−1^of CuO NPs (40 nm) prevented the growth of some oral bacteria [39]. The MIC values of this study were comparable to ours, although the size of our NPs was smaller (18 nm) than theirs.

Further studies are recommended to assess the biocompatibility and mechanical properties of denture liners containing CuO NPs, especially at MIC concentration. The stability and duration of the effectiveness of denture liners containing CuO NPs should be assessed. It is also suggested that the mechanism of antimicrobial effect of CuO NPs in different strains of fungi and bacteria should be evaluated in the future.

## 5. Conclusion

It was concluded that the CuO NPs incorporated soft denture liner is an efficient, practical, and accessible alternative for denture users with an oral microbial infection. The CuO NPs might have the potential as a microbial resistant coating in biomedical devices and limit the spread of some pathogenic microbes.

## Figures and Tables

**Table 1:** Minimum inhibitory concentration (*µ*g mL^−1^) of CuO NPs against *C. albicans* and *Streptococcus* spp. strains.

Species	ATCC/CBS	MIC (*µ*g mL^−1^)
*C. albicans*	C10261	64
*C. albicans*	C1905	64
*C. albicans*	C1912	64
*C. albicans*	C1949	128
*C. albicans*	C2730	64
*S. mutans*	A35668	128
*S. sobrinus*	A27607	128
*S. salivarius*	A9222	64

Note: MIC: minimal inhibitory concentration; ATCC: American Type Culture Collection; CBS: Centraal Bureau Voor Schimmelcultures.

**Table 2:** Biofilm formation of *C. albicans* and *Streptococcus* spp. strains on the soft denture liner disks containing different concentrations of CuO NPs.

Microbial species	Biofilm formation in different concentrations of CuO NPs (%) ± SD				
	0 (*µ*g mL^−1^)	0.5 (*µ*g mL^−1^)	5.0 (*µ*g mL^−1^)	50 (*µ*g mL^−1^)	500 (*µ*g mL^−1^)
*C. albicans* (CBS 10261)	100	87 ± 5.3	72 ± 2.9	50 ± 2.6	25 ± 2.9
*S. mutans* (ATCC35668)	100	95 ± 4.8	82 ± 4.1	66 ± 3.9	70 ± 6.3
*S. sobrinus* (ATCC27607)	100	83 ± 6.1	75 ± 5.1	62 ± 4.1	37 ± 5.1
*S. salivarius* (ATCC9222)	100	90 ± 3.1	89 ± 4.6	65 ± 5.1	40 ± 4.9

Note: ATCC: American Type Culture Collection; CBS: Centraal Bureau Voor Schimmelcultures; SD: standard deviation.

## Data Availability

All the data used to support the findings of this study are available from the corresponding author upon request.

## Additional Points

Scientific rationale for study: the use of soft denture liners is associated with several problems such as difficulty in cleaning, microbial colonization, and subsequently caused denture stomatitis. In old patients, cleansing procedures are challenging and make soft liners susceptible to plaque accumulation. To reduce this problem, several solutions such as adding NPs have been suggested. Principal findings: CuO NPs caused a significant reduction of microbial growth and incorporation of these NPs in soft denture liner decreased biofilm formation. Practical implications: the soft denture liner with CuO NPs is proposed as an appropriate carrier for the prevention of denture stomatitis and improves their properties.
